# Mitochondrial dysfunction in fibrotic diseases

**DOI:** 10.1038/s41420-020-00316-9

**Published:** 2020-09-05

**Authors:** Xinyu Li, Wei Zhang, Qingtai Cao, Zeyu Wang, Mingyi Zhao, Linyong Xu, Quan Zhuang

**Affiliations:** 1 Transplantation Center of the 3rd Xiangya Hospital, Central South University, 410013 Changsha, Hunan China; 2 Xiangya School of Medicine, Central South University, 410013 Changsha, Hunan China; 3 Hunan Normal University School of Medicine, 410013 Changsha, Hunan China; 4 Pediatric Department of the 3rd Xiangya Hospital, Central South University, 410013 Changsha, Hunan China; 5 School of Life Science, Central South University, 410013 Changsha, Hunan China; 6 Research Center of National Health Ministry on Transplantation Medicine, 410013 Changsha, Hunan China

**Keywords:** Mitochondria, Metabolic disorders

## Abstract

Although fibrosis is a common pathological feature of most end-stage organ diseases, its pathogenesis remains unclear. There is growing evidence that mitochondrial dysfunction contributes to the development and progression of fibrosis. The heart, liver, kidney and lung are highly oxygen-consuming organs that are sensitive to mitochondrial dysfunction. Moreover, the fibrotic process of skin and islet is closely related to mitochondrial dysfunction as well. This review summarized emerging mechanisms related to mitochondrial dysfunction in different fibrotic organs and tissues above. First, it highlighted the important elucidation of mitochondria morphological changes, mitochondrial membrane potential and structural damage, mitochondrial DNA (mtDNA) damage and reactive oxidative species (ROS) production, etc. Second, it introduced the abnormality of mitophagy and mitochondrial transfer also contributed to the fibrotic process. Therefore, with gaining the increasing knowledge of mitochondrial structure, function, and origin, we could kindle a new era for the diagnostic and therapeutic strategies of many fibrotic diseases based on mitochondrial dysfunction.

## Facts

- Fibrosis is the major pathophysiologic basis and ultimate pathway for most parenchymatous organ injury.
- Mitochondria play a central role in energy metabolism and even decide the cellular fate.
- Mitochondrial dysfunction could induce fibrotic diseases.
- Targeting mitochondria may help alleviate fibrosis.

## Open questions

- What are the mechanisms of mitochondrial dysfunction in fibrosis?
- How are mitochondria involved in the development of fibrosis?
- How to prevent or slow down fibrosis through targeting mitochondria?

## Introduction

The fibrotic disease is a major health problem worldwide. As the common pathological pathway to organ injury and failure, fibrosis usually represents an unsatisfactory prognosis^1^. It is the major cause of death in the world and causes substantial medical and economic burdens^1,2^.

As the power house of cells, mitochondrion maintains the basic functions of every single cell in our body, including energy metabolism, cell differentiation modulation, signaling transduction and apoptosis^3,4^. Reactive oxygen species (ROS) are byproducts of normal metabolism. The functional mitochondrion has the ability to control the balance of ROS biogenesis and scavenging. However, severe redox stress events will lead to the disruption of homeostasis. Excessive ROS will destroy the normal structure and function of mitochondria and release from mitochondria via mitochondrial permeability transition pore (mPTP) opening mechanism^5^. When mitochondrial dysfunction occurs, the normal cellular biological processes are disrupted, and the oxygen homeostasis is destroyed in tissue.

In the process of fibrosis, the injured tissue shows the characteristics of oxidative stress, hypoxia, and inflammation^6,7^. In this damaging microenvironment, mitochondrial dysfunction usually occurs, which is closely related to the development of fibrotic diseases^8^. This review highlighted the emerging mechanisms related to mitochondrial dysfunction in different fibrotic organs and tissues, including mitochondria morphological changes, mitochondrial membrane potential damage, mitochondrial DNA (mtDNA) damage, ROS production, mitophagy abnormality, mitochondrial transfer, etc. Furthermore, we summarized the therapeutic strategies targeting mitochondria, aiming to provide new clinical therapy for the combined effect of mitochondria.

## Mechanism of fibrotic process

Fibrosis is the terminal development of chronic inflammation in many organs^1^. When tissue damage is severe or repeated beyond the regeneration ability of surrounding parenchymal cells, normal tissue repair becomes excessive, interstitial fibrous connective tissue will repair a large number of proliferation, resulting in pathological changes of fibrosis^6^. It is generally believed that activated fibroblasts act as the key cells that ultimately lead to fibrosis. Activated fibroblasts (i.e. myofibroblasts) express α-smooth muscle actin (α-SMA), increase the expression of fibro-collagen (type I, III, V, and VI) and other extracellular matrix (ECM) macromolecules, and inhibit ECM degrading enzymes^9,10^. Moreover, transforming growth factor-β (TGF-β), a common anti-inflammatory cytokine mainly produced by macrophages, plays a critical role in the fibrosis development^11^, which is an effective inducer of myofibroblasts, and stimulates the expression of key genes in fibrosis through several downstream pathways, especially Smad signaling^11–13^.

Oxidative stress and hypoxia are pretty relevant to fibrosis^6,7^. In fibroblasts, hypoxia could increase ROS production in mitochondria^14^, in which ROS affects the synthesis, secretion, and degradation of ECM. And there is a strong correlation between TGF-β signal transduction and ROS^15,16^.

## Mitochondrial dysfunction

### Normal functions of mitochondrion

Mitochondrion is the power house of cells. As a semi-autonomous organelle, mitochondrion maintains the basic cellular function, including adenosine-triphosphate (ATP) production, ROS biogenesis and scavenging, cell differentiation modulation, signaling transduction, and apoptosis^3,4^. Mitochondrial inner membrane contains enzymes involving electron transport chain (ETC) and ATP production, and electrochemical gradients across the inner membrane drive the process of oxidative phosphorylation (OXPHOS)^17–23^. The energy of most cells in the body is produced by mitochondria through tricarboxylic acid (TCA) cycle and ETC. ETC consists of five subunit enzyme complexes located in mitochondrial inner membrane, including complexes I, II, III, IV, and V^24^.

### Mitochondrial dysfunction

Mitochondrial dysfunction refers to the damage of mitochondrial structure, respiratory chain defects, biogenic dysfunction, gene damage, reduction of mitochondria number and changes in oxidative protein activity in cells and tissues. ROS is a byproduct of oxygen metabolism, and mitochondria have been found to serve as the main source of ROS in mammals. The imbalance between ROS production and removal results in cumulative ROS contacting with mitochondria and cellular components, leading to oxidative damage to mitochondrial proteins, DNA, and lipids^25,26^. The mPTP located in the mitochondrial inner membrane could be open under the conditions of increasing ROS. Mitochondrial permeability transition can induce mitochondrial depolarization and swelling, decrease of ETC activity and release of apoptotic factors^27–29^. In addition, mtDNA lacking histone protection is highly sensitive to ROS and prone to be damaged and mutated under oxidative stress, resulting in respiratory chain defects and decrease of mitochondrial biogenesis^30,31^.

### Mechanisms of mitochondrial self-repair

Meanwhile, mitochondria have multiple mechanisms of self-repair and renewal. Enzymatic defense systems play an antioxidant role such as superoxide dismutase (SOD), catalase (CAT). Mitochondrial biogenesis maintains the number and size of mitochondria. Several transcription factors regulate mitochondrial biogenesis^32,33^. Peroxisome proliferator-activated receptor (PPAR)-γ coactivator-1α (PGC-1α) interacts with many transcription factors/proteins to promote mitochondrial biogenesis and OXPHOS via acting as a transcription co-activator for nuclear receptors^34,35^.

Furthermore, mitochondrial dynamics is a process in which mitochondria form a network through dynamic balance of fission and fusion. Mitophagy can remove dysfunctional mitochondria by fusion with lysosomes^36,37^, thereby controlling the number of mitochondria and maintaining energy metabolism stability^38,39^. ROS can induce mitophagy by activating phosphatase and tensin homology deleted on chromosome 10 (PTEN) induced putative kinase 1 (PINK1)/Parkin pathway^40^. As a ubiquitin kinase, cellular prion protein (PrPc) binds PINK1, enters the mitochondrial inner membrane and is degraded under normal physiological conditions. Under oxidative stress, PINK1 recognizes and aggregates on the surface of damaged mitochondrial extracorporeal membrane, activates phosphorylation, and recruits Parkin translocation. Ubiquitous mitochondria are encapsulated to form mitophagosome, which are fused with lysosomes and reduced by hydrolases^41,42^.

## Mitochondrial dysfunction in solid organ fibrosis

### Mitochondrial dysfunction in cardiac fibrosis

Cardiac fibrosis with ECM deposition can lead to impaired cardiac function and potential heart injury^43^. ROS could directly regulate the production of interstitial ECM by modulating both expression and metabolism of matrix protein^44^. Importantly, the majority of ROS in cardiac fibrosis comes from mitochondria^45^. Therefore, treatment strategies targeting mitochondria are critical. For example, Dai et al. confirmed that the overexpression of antioxidant enzyme CAT targeted to mitochondria, but not wild-type peroxisomal CAT, alleviated mitochondrial oxidative damage, cardiac fibrosis and hypertrophy^46^. Mitoquinone (MitoQ), a mitochondrial-targeted antioxidant, could inhibit fibrosis in pressure overloaded hearts via targeting mitochondrial ROS-mediated signaling TGF-β1, NADPH oxidase 4 (NOX4), and Nrf2 pathway^47^. Bendavia and alogliptin could improve mitochondrial dysfunction, relieved cardiac fibrosis by improving mitochondrial biogenesis^48,49^.

Furthermore, mitochondria in cardiac fibrosis often show diverse dysfunctional forms. The mtDNA lacking histone protection is highly sensitive to ROS. The deletion of mtDNA induced by angiotensin II was reported in cardiac fibrosis of hypertensive cardiomyopathy. Primary damage to mtDNA induced by zidovudine or homozygous mutation of mitochondrial polymerase γ, could also improve cardiac fibrosis^46^. Valli et al. showed that age-dependent cardiac fibrosis was closely associated with mitochondrial dysfunction due to PGC-1β deficiency, a transcriptional regulator of mitochondrial genes^50^.

Nucleotide-binding domain and leucine-rich repeat containing PYD-3 (NLRP3) is a pattern recognition receptor, and it usually responds to inflammation in the form of a multiprotein platform (NLRP3 inflammasome)^51^. Recent studies displayed that NLR family, pyrin domain containing 3 (NLRP3) regulated mitochondrial ROS production in human cardiac fibroblasts. NLRP3 localized to mitochondria regulated myofibroblast differentiation and Smad signal transduction by inducing ROS. Notably, this mechanism is independent of inflammasome. This study indicates the new role of mitochondrial NLRP3 protein involved in fibrosis in non-professional immune cells^52^.

Mitochondrial membrane potential and membrane structural damage are also important characteristics in cardiac fibrosis. It is reported that alogliptin alleviated interstitial fibrosis in diabetic rabbits by reducing the production of mitochondrial ROS, preventing the mitochondrial membrane depolarization, and improving the swelling of mitochondria^49^. Similarly, melatonin and ephedrine-4 could alleviate oxidative stress and cardiac fibrosis through maintaining the integrity of mitochondrial membrane and preventing the release of cytochrome C^53^.

Additionally, the dysfunction of oxidative respiratory chain and metabolic-related enzymes is also emerging as the pivotal mechanism of cardiac fibrosis. Kennedy et al. identified that the deficiency of nuclear-encoded mitochondrial inorganic pyrophosphatase (PPA2) due to biallelic missense mutations was associated with cardiac fibrosis by using whole-exome sequencing, of which mechanism is related to suppress the activity of respiratory chain complex I and IV, and decreased the expression of citrate synthase in fibroblasts^54^. According to the study by Fraccarollo et al. the activation of nitric oxide (NO)/heme-independent soluble guanylate cyclase (sGC) provided protection through increased expression of uncoupling protein 3 (UCP3) and manganese SOD (MnSOD) genes against mitochondrial SOD production and progressive fibrotic remodeling. Ultimately, this process inhibited human cardiac fibroblast differentiation and ECM accumulation^55^. And one of the mitochondrial sirtuins, Sirt4, accelerated Ang II-induced pathological cardiac hypertrophy via suppressing MnSOD activity in cardiomyocytes from transgenic mice^56^. Among them, UCP3 had an effect on ETC, which could decrease protonmotive force and attenuate ROS production through mild uncoupling^57^. And the deletion of MnSOD would have adverse effects on mitochondrial ETC, TCA cycle, mtDNA stability, and iron metabolism^58^.

**Fig. 1:**
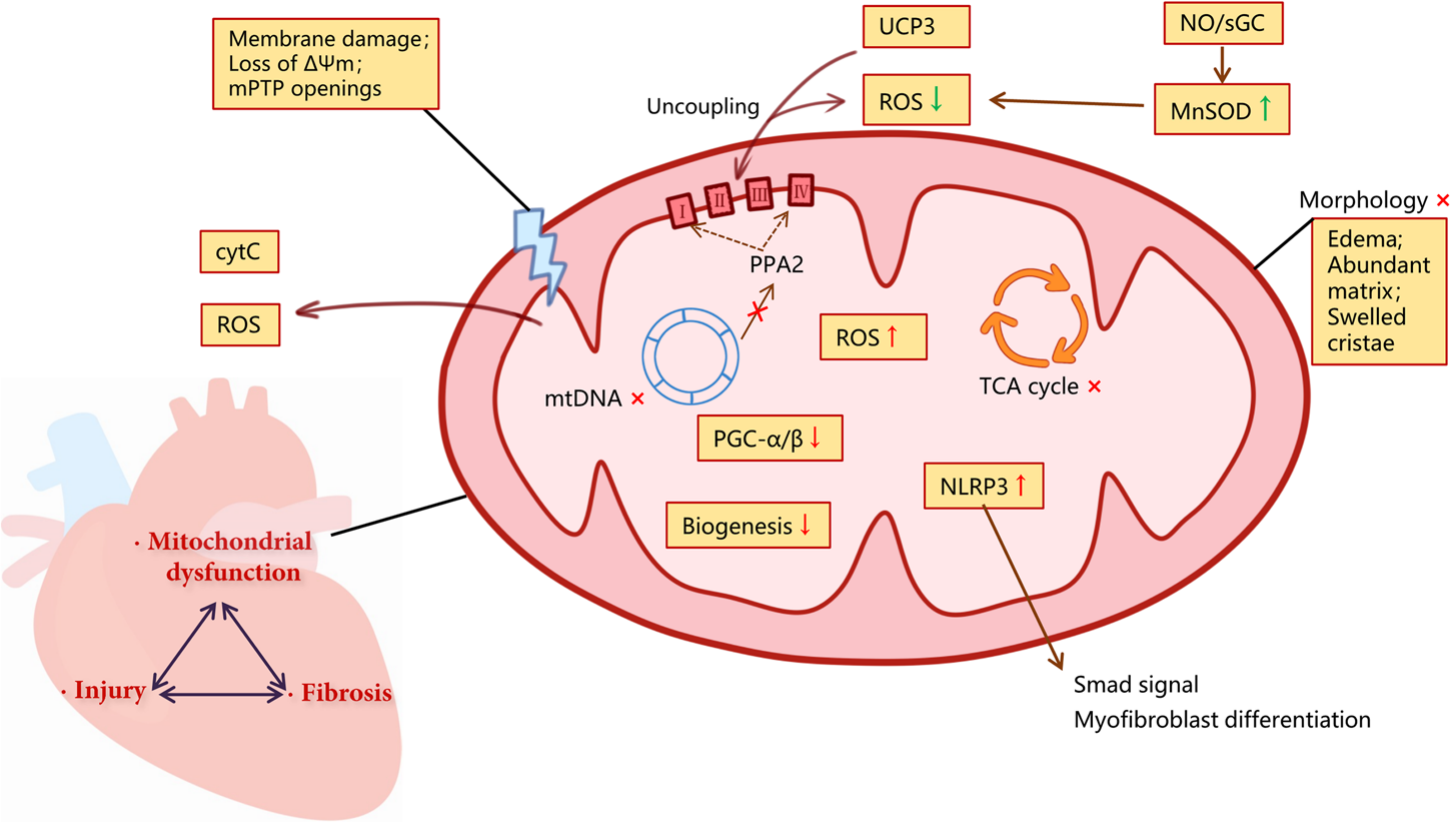
Mitochondrial dysfunction in cardiac fibrosis. Cardiac fibrosis, heart injury, and mitochondrial dysfunction are mutually causal, and the mechanisms overlap. Mitochondrial dysfunction is accompanied by morphological changes, mitochondrial membrane potential, and structural damage, and mtROS production. Excessive mtROS will destroy the normal structure and function of mitochondria, which further leads to the disorder of mitochondrial metabolic function. The release of risk factors like ROS and CytC from mitochondria further aggravates injury and inflammation. Meanwhile, transcriptional regulator deficiency and inhibited mitochondrial biogenesis pathways limit the self-repair function. NLRP3 which was localized to mitochondria regulates myofibroblast differentiation and Smad signal transduction by inducing ROS. As one of the protective pathways, UCP3 and NO/sGC can reduce ROS by mild decoupling and upregulating MnSOD.

We summarized the details of every mechanism above in Fig. 1.

### Mitochondrial dysfunction in pulmonary fibrosis

**Fig. 2:**
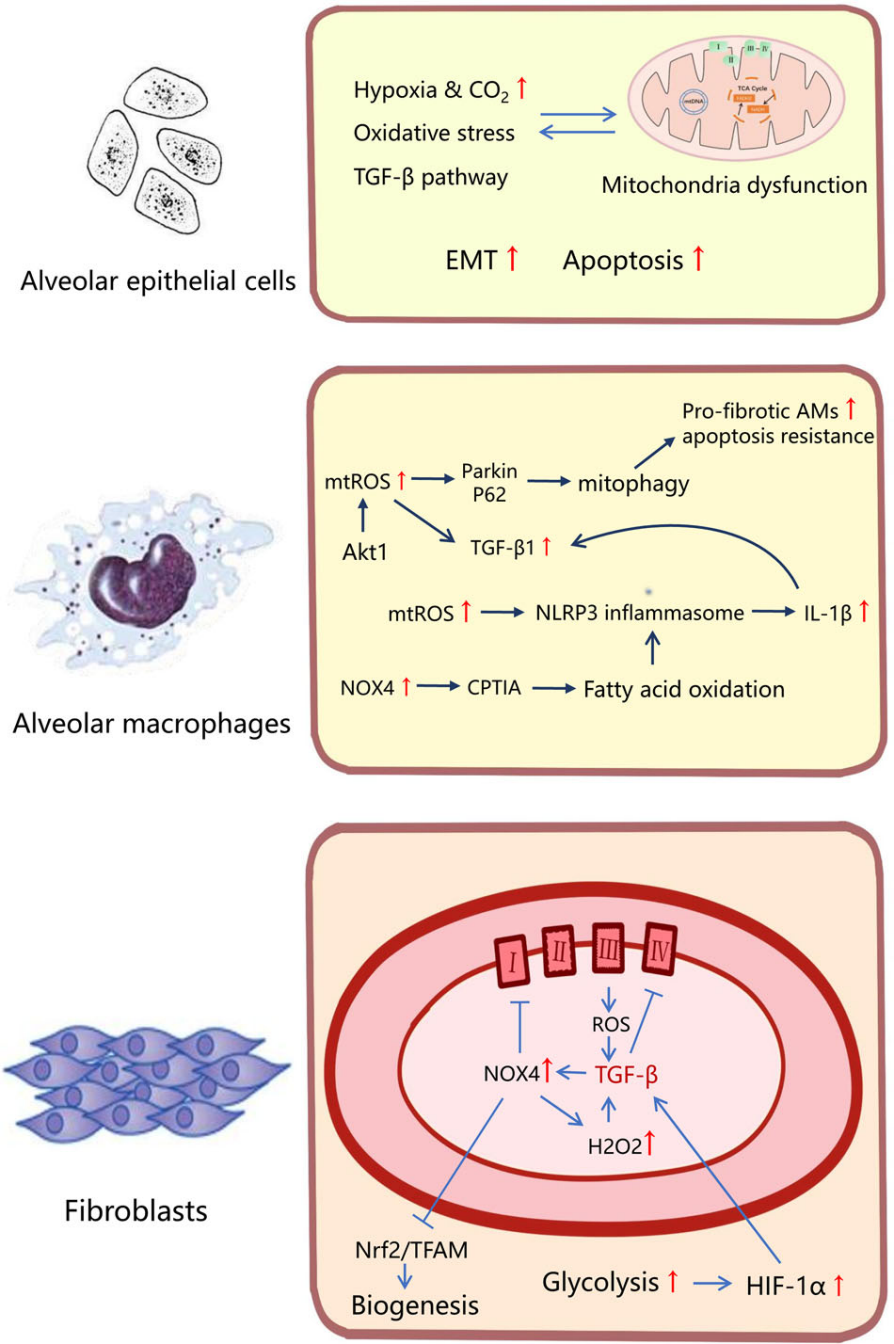
Mitochondrial dysfunction in pulmonary fibrosis. The mitochondrial dysfunction of different cells shows different characteristics in pulmonary fibrosis. The mitochondrial abnormalities and mitochondria-mediated apoptosis in AECs could conduce to pulmonary fibrosis in a critical way. HIF, high level of mtROS and endogenous TGF-β1 signaling interact with apoptosis and EMT. In AMs, Akt1-mediated mtROS could cause mitophagy, which contributed to the apoptotic resistance of pro-fibrotic AMs. As a risk factor in fibrosis, TGF-β1 was activated in response to ROS and NLRP3 inflammasome, which could also induce mitochondrial dysfunction in AMs. The deficiency of NOX4 reduced the mitochondrial fatty acid oxidation, which could inhibit NLRP3 inflammasome activation. ROS produced by complex III were required for TGF-β to induce gene expression in human lung fibroblasts. In turn, TGF-β could also increase the ROS level through the mechanism like inhibition of complex IV. Moreover, generation of H_2_O_2_ dependent on NOX4 was demanded for myofibroblast differentiation induced by TGF-β. Furthermore, the metabolic reprogramming in myofibroblast shows a augmented glycolysis, which contributed to pulmonary fibrosis via promoting the stabilization of HIF-1α.

Pulmonary fibrosis, a pathological change in the development of various and age-related end-stage lung diseases, generally is featured by not only activation and proliferation of fibroblast accompanied by tissue damage and inflammation, but also increased deposition of mesenchymal collagen^59^. The vast majority of patients with pulmonary fibrosis have an unknown cause (idiopathic), defined as idiopathic interstitial pneumonia (IIP) or idiopathic pulmonary fibrosis (IPF)^60^. Recently, there is growing evidence that mitochondrial dysfunction may contribute to the pathogenesis of IPF. We have sketched Fig. 2 to summarize the key information of every mechanism below.

The mitochondrial abnormalities and mitochondria-mediated apoptosis in alveolar epithelial cells (AECs) could conduce to pulmonary fibrosis in a critical way. Mitochondrial ROS, always with an increased level, mediated by a variety of mechanisms. Hypoxia and high CO_2_ level can decrease oxygen consumption and ATP production in AECs and impair cell proliferation through mitochondrial ROS^61,62^. Furthermore, EMT of AECs was induced by hypoxia through hypoxia inducible factor (HIF), high level of mitochondrial ROS, and endogenous TGF-β1 signaling^61^. It was reported that oxidative stress induced preferential mtDNA damage in a variety of AECs. Sirtuin (silent mating type information regulation 2 homolog) 3 (SIRT3) deficiency could improve lung fibrosis by augmenting apoptosis and mtDNA damage in AECs^63^. As a key enzyme for base excision repair with the function of alleviating pulmonary fibrosis, 8-oxoguanine DNA glycosylase (Ogg1) associated with aconitase-2 (Aco-2) could prevent mtDNA damage, p53 mitochondrial translocation, and intrinsic apoptosis in AECs^64^. Furthermore, thyroid hormone could increase biogenesis via activating PGC-1α and promote mitophagy via PINK1 in mice, which helped suppress mitochondria-mediated apoptosis and reversing bleomycin-induced mitochondrial abnormalities in AECs^65^.

Compared with the normal lung fibroblasts, there had been shown that not only a decrease of mitochondrial mass and morphologic alteration, but also the low level of oxygen consumption rate and ATP production in the ones of pulmonary fibrosis^66^. Excessive TGF-β pathway could result in robust profibrotic gene expression in fibroblasts, leading to fibrosis. It was confirmed that ROS produced by complex III were required for TGF-β to induce gene expression in primary normal human lung fibroblasts^67^. TGF-β could also directly or indirectly increase the ROS level through various mechanisms, such as inhibition of complex IV and activation of NADPH oxidase^61^. Moreover, generation of hydrogen peroxide (H_2_O_2_) dependent on NOX4 was demanded for myofibroblast differentiation, which was induced by TGF-β1^68^. Also, lysocardiolipin acyltransferase (LYCAT) could protect against pulmonary fibrosis through negatively modulating TGF-β-induced lung fibroblast differentiation via the decline of NOX-dependent H_2_O_2_ generation and mitochondrial superoxide^69^. In addition, augmented glycolysis contributed to pulmonary fibrosis via promoting the stabilization of HIF-1α in myofibroblast, which could increase the expression of TGF-β1 and regulate the glycolytic enzymes^70^. In lung fibroblasts, NOX4 inhibited mitochondrial bioenergetics and biogenesis through decreasing induction and activation of endogenous nuclear factor (erythroid-derived-2)-like-2 factor (Nrf2) and mitochondrial transcription factor A (TFAM) or directly inhibiting complex I of ETC, whereas inactivation of TORC1/PGC-1 axis could repress mitochondrial biogenesis and bioenergetics via downgrading the expression of Nrf1 and TFAM^71,72^. Metformin could attenuate lung fibrosis development through NOX4 inhibition. Activation of AMPK mediated by metformin inhibits NOX4 expression induced by TGF-β^73^. It was reported that AMPK activity was lower in fibrotic regions. AMPK-deficient fibroblasts reduced basal oxygen consumption, diminished mitochondrial reserve capacity and maximal respiration^74^. In a bleomycin model, metformin could reverse the established lung fibrosis. Metformin could active AMPK reprogramming metabolism of IPF fibroblasts via diminishing mTOR activation and promoting autophagy, as well as downregulating homeostasis levels of ECM proteins. AMPK activation also upregulated mitochondrial biogenesis. Furthermore, it restored myofibroblast sensitivity to intrinsic apoptosis, particularly induced by antimycin A, a mitochondrial inhibitor of ETC complex II^74^.

Importantly, there was evidence of vital role of alveolar macrophages (AMs) in the process of pulmonary fibrosis. Dioscin could alleviate crystalline silica-induced excessive mitochondrial ROS release, AMs apoptosis, and mitochondrial dysfunction, such as MMP depolarization and low ATP production. Dioscin promoted AMs autophagy, decreasing production of inflammatory factors in vivo and in vitro, thereby reducing collagen deposition and inflammatory infiltration^75^. In AMs, Akt1-mediated mitochondrial ROS could cause mitophagy, which contributed to the apoptotic resistance of pro-fibrotic AMs, expression of TGF-β1 and activation of myofibroblasts^76^. TGF-β1 could induce mitochondrial dysfunction in AMs, such as suppression of the OXPHOS, low level of mitochondrial ATP production, and MMP depolarization^77^. Notably, the ROS production in AMs induced NLRP3 inflammasome activation, which was related to mitochondrial dysfunction^78^. And in the mouse model of NLRP3-mediated *Streptococcus pneumoniae* infection, the deficiency of NOX4 reduced the mitochondrial fatty acid oxidation, which could inhibit LRP3 inflammasome activation and improved survival^5^. The mechanisms of mitochondria involved in NLRP3 inflammasome activation further suggested that mitochondria may play a critical role in chronic inflammation.

### Mitochondrial dysfunction in renal fibrosis

Renal fibrosis is the formation of scars in the parenchyma, which is the commonly accepted to serve as ultimate pathway for almost all chronic and progressive nephropathy^1^. Like other organs, decreased expression of Gα-binding protein (GABP), PGC-1α, and PPAR-α indicated a decrease of mitochondrial biogenesis in fibrosis^79^. Additionally, the integrity of mitochondrial morphology and structure was often destroyed in renal fibrosis^80^. It was reported that melatonin prevented mitochondrial edema, cristae dilatation and maintained the integrity of mitochondrial membrane, thereby alleviating renal fibrosis^8^. TNF receptor-associated protein 1 (TRAP1) could inhibit the fibrosis-related proteins expression in renal tubular epithelial cells (TECs) and tubulointerstitial fibrosis by alleviating mitochondrial vacuolation, swelling, matrix density reduction and mitochondrial cristae rupture, and increasing the number of mtDNA copies^81^.

Moreover, the changes in mitochondria permeability could lead to the release of cytochrome C and other substances, which mediated apoptosis^82^. The oxidative damage of cardiolipin-sensitized mitochondria to calcium, induced mitochondrial permeability transition and destroyed the permeability barrier of IMM, which caused the collapse of mitochondrial potential, the decoupling of OXPHOS and apoptosis, and the release of cytochrome C to cytosol^83,84^. A cell-permeable peptide Bendavia (SS-31) targeted the inner mitochondrial membrane and binded to cardiolipin, which could protect mitochondria in medullary TECs^83^. Bendavia improved oxidative stress and tubulointerstitial fibrosis and restored renal vascular endothelial function in vivo and in vitro^84^. The anti-fibrotic mechanism was to reduce oxidative damage of mitochondrial cardiolipin.

According to the study of Zhang et al.^85^ after renal ischemia reperfusion injury (IRI), ROS production and mtDNA damage increased, which could lead to EMT and further renal fibrosis. Postconditioning (POC) therapy can reduce renal fibrosis by protecting mitochondria from oxidative stress-induced mtDNA damage. Furthermore, in renal fibrosis and renal injury induced by AAI, decrease of mtDNA-encoded cytochrome C oxidase subunit 1 (COX-1) and nuclear DNA (nDNA)-encoded nicotinamide adenine dinucleotide dehydrogenase (ubiquinone)-1b subcomplex 8 (NDUFb8) suggested that mtDNA and nDNA were both victims. However, with the progression of aristolochic acid nephropathy (AAN) in renal cortex, NDUFb8 level restored, while COX-1 level maintained low^80^. Similarly, the activity of respiratory complex 1, which is partly encoded by mtDNA, was more significantly impaired than that of respiratory complex II, completely encoded by nDNA^86^. These results suggested that mtDNA damage would be more severe than nDNA damage on fibrosis.

In terms of oxidative respiratory chain and metabolic enzymes, cardiolipin peroxidation disrupts respiratory chain complex and inhibits mitochondrial respiratory compounds^83,84^. As mentioned before, COX-I and NDUFb8 defects also played critical roles in respiratory chain damage^80^. Moreover, Ang-II induced renal injury by alleviating mitochondrial dysfunction, which was related to the decrease of ATP synthase activity, 1,25-dihydroxyvitamin D3 alleviated this situation^87^.

NLRP3 could be considered as another important risk factor. It is reported that activated NLRP3 inflammasome is involved in kidney injury process via mitochondrial dysfunction in TECs and macrophages. NLRP3 caused mitochondrial dysfunction, increased ROS and finally led to fibrosis^88,89^. However, studies also showed that NLRP3 promoted the TGF-β/Smad signaling pathway in TECs independent of the inflammasome^90^. In renal tubular cells, NLRP3 transferred from the cytosol to the mitochondria and targeted to mitochondrial antiviral signal protein (MAVS) during hypoxia, which played a critical role in mitochondrial ROS accumulation and disfunction^91^. Therefore, the deletion of NLRP3 is emerging as a potential therapeutic target, which can attenuate fibrosis via protecting the damaged mitochondrial function.

During the treatment of chronic kidney disease (CKD) based on mesenchymal stem cells (MSCs), MSCs in patients with CKD experienced accelerated aging and suppressed efficacy. It is reported that melatonin enhances the role of MSCs in CKD treatment and alleviates fibrosis, which improves mitochondrial function through high expression of PrPc. High expression of PrPc can increase the activity of complexes I and IV, thereby enhancing OXPHOS of mitochondria. Moreover, PINK1 could promote mitochondrial dynamics and metabolism^92^.

### Mitochondrial dysfunction in hepatic fibrosis

Hepatic fibrosis is the major pathophysiologic basis and final common pathway of various chronic hepatic diseases, such as alcoholic liver disease, viral infection and non-alcoholic steatohepatitis (NASH)^93^. In the process of liver injury^94^, HSCs transform from static physiological state to fibrotic phenotype^95,96^. This transformation is induced by inflammatory mediators, ROS and apoptotic bodies arising from dying hepatocytes and activated HSCs. Growing evidence supported that the hepatic fibrosis via HSCs activation was associated with mitochondrial dysfunction.

According to the chemiosmotic theory, mitochondrial electron transfer is accompanied by proton flux and coupled by redox proton pump mediated by mitochondrial complexes (CI, CIII, and CIV). Mitochondrial uncouplers can make the energy generated by electron transfer in the respiratory chain not be used for the phosphorylation of ADP, but can only be emitted in the form of heat. A recent study suggested that mitochondrial uncouplers could inhibit HSCs activation via reducing ATP and ROS level^97^.

Augmenter of liver regeneration (ALR) is a hepatocyte survival factor induced by mitochondrial dysfunction/damage and cell death upon inhibition of its synthesis. Ai et al. found that inhibition of ALR expression aggravated hepatic fibrosis, probably through enhancing mitochondrial fusion and HSCs migration. In HSCs, ALR could induce the mitochondrial Ca^2+^ influx increase, which attributed to the HSCs migration. ALR transfection retarded HSCs migration and suppressed F-actin assembly, while promoting mitochondrial fission and diminishing ATP synthesis^98^. ALR gene therapy, which has been shown to improve the hepatic fibrosis effectively, could inhibit the ATP loss, reduce intrahepatic ROS level, enhance the activity of ATPase, and decrease expression of TGF-β1, PDGF, and α-SMA^99^.

NLRP3 inflammasome induces caspase 1-dependent release of proinflammatory cytokines IL-1 β and IL-18, which induce cell death under inflammatory and stress conditions. As one of many important NLRP3 inflammasome activators, ROS have been reported to promoting the chronic liver disease, including hepatic fibrosis. Especially in HSCs, the up regulation of NOX4 expression, which is a producer of ROS, has been found to be related to the activation of NLRP3 inflammatory and the increase of collagen production. Didymin can notably ameliorate chronic hepatic injury and collagen deposition, with inhibition of HSCs proliferation and induction of apoptosis, and it also significantly causes mitochondrial membrane depolarization, usually accompanied by cytochrome C release in HSCs. Didymin can improve the hepatic fibrosis mainly by inhibition of ERK/MAPK and PI3K/Akt pathways through increasing Raf kinase inhibitor protein (RKIP) expression in HSCs^100^. p66Shc, a redox enzyme that regulates mitochondrial ROS generation, contributes to hepatic fibrosis, whereas its inhibition can ameliorate the liver fibrosis through restraining the activation of HSCs via down-regulating mitochondrial ROS production and NLRP3 expression^101^.

For mitochondrial homeostasis and normal ATP level, it is critical to keep the normal ETC and normal activity of enzyme related to respiratory chain. Poly (ADP-ribose) polymerase (PARP) is a key mediator of liver fibrosis, and its inhibition or genetic deletion can protect against hepatic fibrosis via ameliorating the abnormal ETC and improving the activation of complex I and IV^102^. Enzyme activity related to respiratory also plays a very important role in the entire unit. Nabanita et al. found that melatonin could ameliorate hepatic fibrosis via restoring the enzymatic activities associated with respiratory chain, decreasing mitochondrial ROS production and inhibition of HSCs activation^103^. As we already know, the activation of HSCs around hepatic sinusoids is the main source of liver fibrosis in any etiology. So promoting HSCs apoptosis is a strategy worth considering. Chen et al. found that dihydroartemisinin prevented liver fibrosis through promoting HSCs apoptosis via down-regulating the PI3K/Akt pathway. Dihydroartemisinin could induce HSCs apoptosis via promoting loss of mitochondrial transmembrane potential (MTP) in HSCs, transfer of cytochrome C from mitochondria to cytoplasm, and the decreased ratio of anti-apoptotic BCL-2 to pro-apoptotic Bax.

Mitochondrial autophagy is a specific selection process, which is precisely regulated by various factors such as PINK1, Parkin, and so on. It is an important regulatory mechanism for cells to clear damaged mitochondria and maintain their homeostasis. Qiu et al. found that PM2.5 induced liver fibrosis through triggering mitophagy mediated by ROS. PM2.5 could induce mitophagy through up-regulating PINK1/Parkin signal pathway via increased ROS, and thus activate HSCs^104^. Additionally, melatonin could protect against liver fibrosis via upregulating mitophagy and mitochondrial biogenesis in mice^105^. In addition, NLRP3 inflammasome activated by NOX4-independent ROS could induce pro-inflammatory factors, including IL-1β, which increased chronic liver inflammation and promoted activation of HSCs. Cai et al. found that angiotensin-(1–7) improved hepatic fibrosis via modulating the NLRP3 inflammasome through redox balance regulation including upregulation of GSH, Nrf2, antioxidant response element (ARE), and down-regulation of hydrogen peroxide, NOX4^106^. In hepatic cells, the overexpression of BCL-2, which is the anti-apoptotic protein with the function of inhibiting hepatic cells apoptosis, can delay fibrosis progression via maintaining the normal ROS level^107^.

**Fig. 3:**
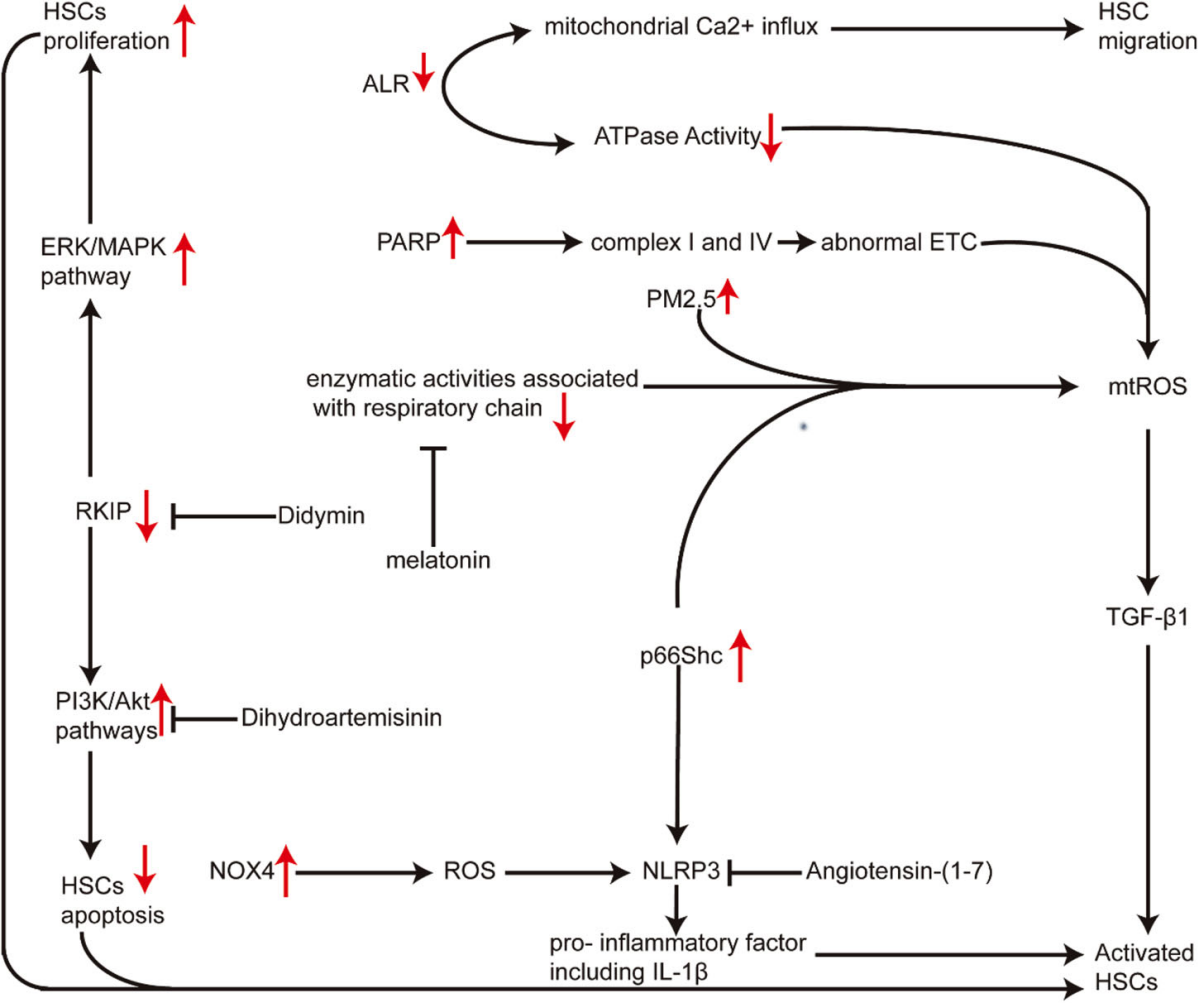
Mitochondrial dysfunction in hepatic fibrosis. The proliferation and activation of HSCs is the central process during the development of HF. Inhibition of ALR expression aggravates liver fibrosis, probably via promoting HSC migration and mitochondrial fusion. The increased mitochondrial Ca^2+^ influx induced by ALR in HSCs attributes the HSC migration. The activation of PARP can aggravate hepatic fibrosis via deteriorating the abnormal ETC including the inhibition of complexes I and IV. p66Shc can contribute to hepatic fibrosis through the activation of HSCs via upregulating mtROS production and NLRP3 expression. Didymin can improve the hepatic fibrosis main by inhibition of ERK/MAPK and PI3K/Akt pathways via up-regulation of RKIP expression in HSCs. The NLRP3 inflammasome activated by NOX4-independent ROS can mediate activation of HSCs via inducing pro-inflammatory factor including IL-1β.

Figure 3 shows the keynotes of all the mechanisms above.

## Mitochondrial dysfunction in tissues

### Mitochondrial dysfunction in skin fibrosis

Radiation‐induced dermatitis can cause skin fibrosis, and radiation also damages mitochondria^108^. Radiation-induced subcutaneous fibrosis can also be associated with genetic variation of thioredoxin reductase 2 (Txnrd2), a mitochondrial enzyme involved in removal of ROS^109^. This reveals that the lost control of ROS clearance and production in mitochondria will lead to serious consequences. JP4-039 is a ROS scavenger with the significant affinity for mitochondrial inner membrane. Topical JP4-039 could prevent skin damage and fibrosis from radiation^110^.

Treatment that selectively induces apoptosis of myofibroblasts could reverse established fibrosis, like scleroderma^111,112^. In fact, the apoptosis induction targeting mitochondria has been gradually applied in the fibrosis treatment. It is reported that increasing of the mitochondrial priming could promote myofibroblast activation, which primed by proapoptotic BH3-only protein BIM. But meanwhile, the antiapoptotic protein BCL-X_L_ sequestered BIM to ensure myofibroblast survival. The “BH3 mimetic” drug (ABT-263) can induce myofibroblasts apoptosis through inhibiting BCL-X_L_^113^.

### Mitochondrial dysfunction in islet fibrosis

**Fig. 4:**
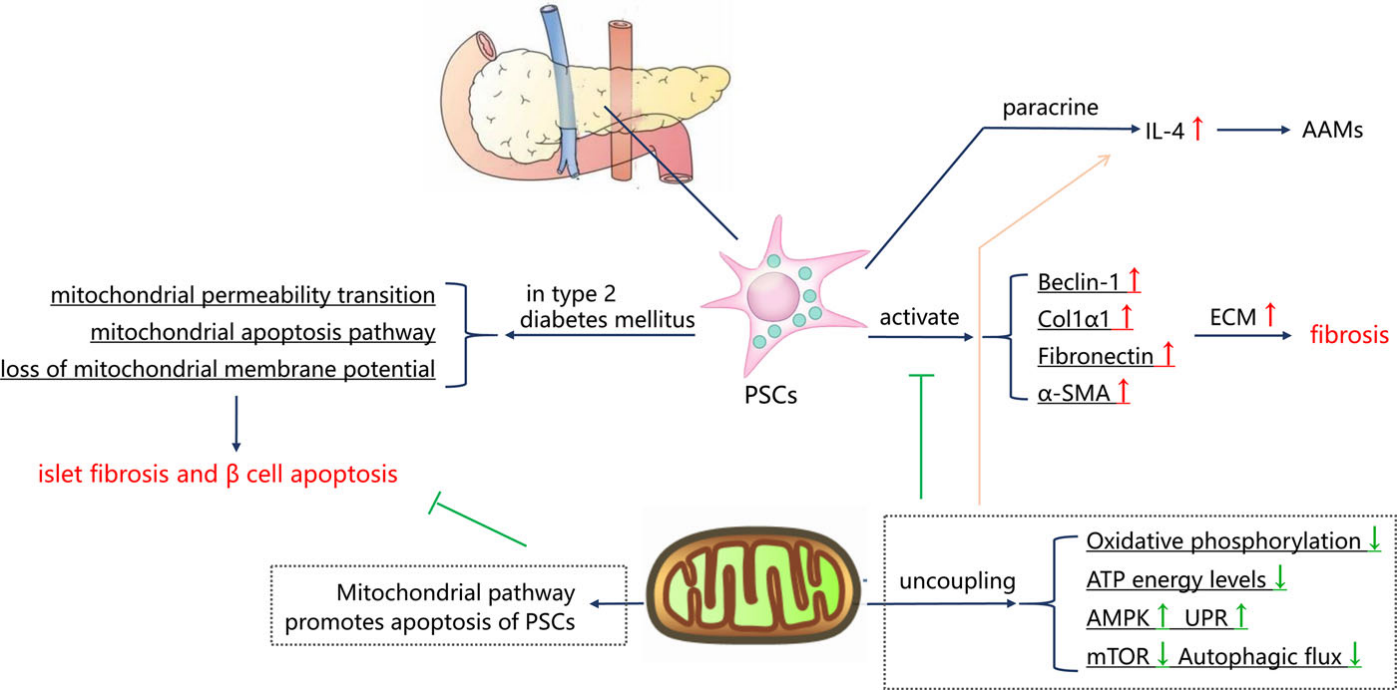
Mitochondrial dysfunction in islet fibrosis. Activated PSCs play a critical role in the remodeling of peripheral ECM, which mediates apoptosis and islet fibrosis by inducing mitochondrial dysfunction of islet cells. Selectively inducing PSCs apoptosis via mitochondrial pathway is a feasible strategy. Furthermore, activated PSCs mainly relies on oxidative phosphorylation of mitochondria to maintain ATP energy levels. The uncoupling of mitochondria decreases oxidative phosphorylation and ATP level to inhibit PSCs activation. But this low cell energy situation can promote the phenotype transformation of AAMs through IL-4 secretion.

Activated pancreatic stellate cells (PSCs) regulates the remodeling of peripheral ECM and plays a paracrine role in adjacent cells. Activated PSCs mainly relies on OXPHOS of mitochondria rather than glycolysis to maintain ATP energy levels and sustained energy-dependent processes^114,115^. Rottlerin acts as an OXPHOS uncoupling agent of mitochondria, which can rapidly depolarize mitochondria, reduce mitochondrial mass, change dynamics, decrease ATP level, activate AMP-activated protein kinase (AMPK), unfolded protein response (UPR) signaling transduction, inhibit mTOR pathway, and block autophagic flux. Therefore, rottlerin reduces the expression of α-SMA and other ECM proteins in PSCs^115^. In addition, we have known that alternatively activated macrophages (AAMs) depend on IL-4 signal transduction, and PSCs are the source of IL-4^116^. Interestingly, the increased expression of IL-4 induced by rottlerin indicates that IL-4 is more easily expressed under low cell energy^115^. This suggests that metabolic reprogramming of PSCs may also play an immunomodulatory role in fibrotic microenvironment. Moreover, PSCs promote islet fibrosis and β cell apoptosis in type 2 diabetes mellitus. The mechanism is that PSCs cause mitochondrial dysfunction, including loss of mitochondrial membrane potential, mitochondrial permeability transition, and mitochondrial apoptosis^117^. Treatment that selectively induces PSCs apoptosis is a feasible strategy, like Tocotrienol can selectively induce the death of PSCs by targeting MTP^118^. An overview landscape of all the mechanisms above is shown in Fig. 4.

## Mitophagy and fibrotic disease

Mitophagy can remove damaged mitochondria and alleviate mitochondrial dysfunction in order to inhibit the development of fibrosis. The defects of mitophagy in fibrosis has been widely reported. In animal models of renal vascular hypertension, mitophagy was inhibited, such as Parkin, LC3-II, ATG5 protein deficiency, accompanied by renal fibrosis^119^. Inhibition of mitophagy activates platelet-derived growth factor receptor (PDGFR)/PI3K/AKT signaling pathway and increases ROS production, accompanied by enhanced differentiation and proliferation of myofibroblasts. Inhibitory mechanism of mitophagy involves the knockdown of PARK2 and the low expression of BECN1 induced by microRNA-1224–5p^120–122^. It is also reported that TGF-β induces the production of ROS and mitochondrial depolarization in pulmonary epithelial cells. However, TGF-β can stabilizes the key mitophagy initiating factor PINK1 on the surface of mitochondria, abrogates ROS, prevents cell death, which is necessary to limit fibrosis^123^. So, the appropriate mitophagy activation in fibrosis may be regarded as a cell self-protection mechanism.

Reducing mitochondria dysfunction by targeting mitophagy has the potential therapeutic value in fibrosis. Melatonin inhibits liver fibrosis by up-regulating PINK, increasing autophagy flux, and upregulating light chain-3 (LC3-II) degradation^105^. PINK1-mediated mitophagy reduces the accelerated aging of MSCs in patients with CKD and enhances the therapeutic effect of transplanted MSCs on renal fibrosis^92^. Interestingly, when mitophagy is excessive, it also mediates the initiation of apoptotic processes. BCL-B belongs to the BCL-2 protein family, inhibits autophagy by binding Parkin signal and inhibiting its phosphorylation. BCL-B knockdown activates mitophagy, promotes apoptosis of HSCs and prevents fibrosis^124^. However, self-renewal mediated by mitophagy is not always beneficial to alleviating fibrosis. Akt1 produces apoptotic resistance in IPF AMs by inducing increased ROS and mitophagy, and increasing the expression of TGF-β1 to promote pulmonary fibrosis^76^. PM2.5 activates PINK1/Parkin pathway by inducing excessive ROS to trigger mitophagy, which activates LX-2 cells and primary HSCs^104^.

## Mitochondrial transfer and fibrosis

It is believed that mitochondrial transfer as a new mode of cell–cell communication can effectively replace defective mitochondria^125^. There are many different ways to transfer mitochondria, including microinjection, incubation with intact purified mitochondria, gap junction channel-mediated cell attachment, and direct transfer from donor cells such as MSCs^125,126^. Extensive studies have confirmed that mitochondrial transfer plays a protective role in diverse organs^127,128^. We have seen burgeoning interest in the relationship between mitochondrial metastasis and fibrosis. MSCs directly transfer mitochondria to receptor cells through spontaneously generated cytoplasmic bridges called tunnel nanotubes (TNT)^129^. Li et al. documented that intravenous injection of bone-marrow-derived MSCs (BM-MSCs) suppressed cigarette smoke (CS)-induced pulmonary fibrosis. However, it is notable that the treatment and mitochondrial transfer to co-cultured bronchial epithelial cells of induced pluripotential cell-derived MSCs (iPS-MSCs) were more effective^130^. In addition, renal scattered tubular cells (STC-like cells)-extracellular vesicles (EV) have capacity for repairing injured TECs and decreasing interstitial fibrosis, partly through transferring STC-like cells functional mitochondria^131^.

As mitochondrial transfer is relatively new research direction, there is still a gap to be filled in fibrotic field. The mechanism, function, and potential clinical application of mitochondrial transfer in fibrosis need and deserve further investigation.

## Therapeutic strategies targeting mitochondria to alleviate fibrosis

In Table 1, we made a summary of therapies targeting mitochondrial dysfunction, which had been mentioned in each organ and tissue section above. These treatment ideas can be summarized as follows: (1) maintain the integrity of the mitochondrial membrane and prevent the release of pro-inflammatory or pro-apoptotic substances; (2) enhance mitochondrial self-repair ability, such as mitophagy, mitochondrial biogenesis; (3) reduce oxidative damage to mitochondrial structure, like mtDNA and cardiolipin; (4) inhibit oxidative stress through exogenous ROS scavenger; (5) restore mitochondrial function or increase the quantity of normal mitochondria through exogenous carriers like MSCs. We also found that melatonin and some small molecular peptides showed therapeutic effects combined with diverse mechanisms. Moreover, cell therapy gradually presents its amazing potential for repair and treatment.

**Table 1:** Therapies targeting mitochondrial dysfunction to alleviate fibrosis.

Organ/disease	Therapeutic strategies	Mechanism	Reference
Heart/ventricle diastolic dysfunction	Alogliptin (a dipeptidyl peptidase-4 inhibitor)	1. Preventing the production of mitochondrial ROS and mitochondrial membrane depolarization; 2. Improving mitochondrial biogenesis by PGC-1α/NRF1/Tfam pathway.	^49^
Heart/cardiorenal syndrome	Melatonin and ephedrine-4	Alleviating oxidative stress, maintaining the integrity of mitochondrial membrane and preventing the release of cytochrome C	^53^
Heart/heart failure	Mitoquinone (a mitochondrial-targeted antioxidant)	1. Inhibiting TGF-β1 and NOX4 expression; 2. Preventing Nrf2 downregulation and activation of TGF-β1-mediated profibrogenic signaling in cardiac fibroblasts	^47^
Heart/hypertensive cardiomyopathy	Overexpress catalase targeted to mitochondria	Alleviating cardiac hypertrophy, fibrosis, and mitochondrial damage	^46^
Heart/renovascular hypertension	Bendavia (a mitochondrial targeted peptide)	Reducing oxidative stress through improving mitochondrial biogenesis	^48^
Lung	8-oxoguanine DNA glycosylase (Ogg1) and aconitase-2 (Aco-2)	Preventing mtDNA damage, p53 mitochondrial translocation, and intrinsic apoptosis in alveolar epithelial cells	^64^
Lung	Thyroid hormone	Increasing biogenesis via activating PGC-1α and promote mitophagy via PINK1	^65^
Lung	Lysocardiolipin acyltransferase (LYCAT)	Negatively modulating TGF-β-induced fibroblast differentiation via the decline of NOX-dependent H_2_O_2_ generation and mitochondrial superoxide	^69^
Lung	Metformin	1. Activation of AMPK mediated by metformin inhibits NOX4 expression induced by TGF-β; 2. AMPK activation also upregulates mitochondrial biogenesis and restores myofibroblast sensitivity to intrinsic apoptosis; 3. AMPK activation reprograms metabolism of IPF fibroblasts via diminishing mTOR activation and promoting autophagy	^73^
Lung	BM-MSCs transplantation	MSCs directly transfer mitochondria to receptor cells through spontaneously generated cytoplasmic bridges called tunnel nanotubes	^130^
Renal	Melatonin	Maintaining the integrity of mitochondrial morphology and structure	^8^
Renal	TNF receptor-associated protein 1 (TRAP1)	1. Maintaining the integrity of mitochondrial morphology and structure; 2. Increasing the number of mtDNA copies	^81^
Renal	Bendavia	Reducing oxidative damage of mitochondrial cardiolipin	^83,84^
Renal/renal ischemia reperfusion injury	Postconditioning therapy	Protecting mitochondria from oxidative stress-induced mtDNA damage	^85^
Renal	1,25-dihydroxyvitamin D3	Maintaining the ATP synthase activity	^87^
Renal	Deletion of NLRP3	Alleviating oxidative stress and ROS production	^88–91^
Renal	Combined treatment of MSC and melatonin	1. Melatonin enhanced the role of MSC in fibrosis treatment; 2. Melatonin improved MSC mitochondrial function and enhanced oxidative phosphorylation through high expression of PrPc; 3. PINK1-mediated mitophagy reduces the accelerated aging of MSCs in patients with CKD and enhances the therapeutic effect	^92^
Renal	STC-like cells-extracellular vesicles	Transferring STC-like cells functional mitochondria to repair injured TECs	^131^
Liver	Augmenter of liver regeneration (ALR) gene therapy	Improving the mitochondrial dysfunction, inhibiting oxidative stress, and suppressing activation of HSCs	^98,99^
Liver	Didymin	Inhibition of ERK/MAPK and PI3K/Akt pathways in HSCs	^100^
Liver	Melatonin	1. Restoring the enzymatic activities associated with respiratory chain, decreasing mitochondrial ROS production and inhibition of HSCs activation; 2. Upregulating mitophagy and mitochondrial biogenesis in mice	^103^
Liver	Dihydroartemisinin	Promoting HSCs apoptosis via mitochondrial pathway and down-regulating PI3K/Akt	^105^
Liver	PARP inhibition	Ameliorating the abnormal ETC and improving the activation of complexes I and IV	^102^
Liver	Melatonin	Up-regulating PINK1 and down-regulating LC3-II/LC3 ratio to promote mitophagy	^105^
Liver	Deletion of BCL-B	Activates excessive mitophagy to promote apoptosis of HSCs	^124^
Skin/radiation-induced skin injury	JP4-039 (a mitochondrially targeted antioxidant)	1. Acting as a ROS scavenger with the significant affinity for mitochondrial inner membrane; 2. Reducing apoptosis and preserving the skin’s antioxidant capacity	^110^
Skin/scleroderma	ABT-263 (a “BH3 mimetic” drug)	Inducing myofibroblasts mitochondrial apoptotic pathway through inhibiting BCL-XL	^113^
Islet	Rottlerin (an oxidative phosphorylation uncoupling agent)	Inhibiting PSCs activation by acting as an oxidative phosphorylation uncoupling agent	^115^
Islet	Tocotrienol	Selectively inducing the death of PSCs	^118^

## Concluding remarks

Mitochondrial dysfunction has long been thought to be closely related to the progression of fibrosis in many end-stage viscera diseases. Mitochondrial dysfunction can lead to changes in mitochondrial morphology, dynamics, metabolic pathways, mtDNA, increased oxidative stress and other harmful substances, and ultimately exacerbate the biogenesis and development of fibrosis. Additionally, the abnormality of mitophagy and mitochondrial transfer also played vital roles in the fibrotic process. Therefore, understanding the process and mechanism of mitochondrial dysfunction is of great therapeutic value for diseases characterized by fibrosis as their pathological feature. With our comprehending of mitochondrial structure, function, and origin in recent years, the diagnosis and treatment of many fibrosis diseases are expected to find a breakthrough in mitochondria.
